# Supplementary material for: The parenting hub of the hypothalamus is a focus of imprinted gene action
Source: PLoS Genet. 2023 Oct 19;19(10):e1010961. doi: 10.1371/journal.pgen.1010961 (PMC10586610; doi:10.1371/journal.pgen.1010961)
Supplement: S1 Text — Whole Hypotahlamus dataset. Figs 1–6 in S1 Text; Tables A-H in s S1 Text. (DOCX) [file pgen.1010961.s001.docx]

**The parenting hub of the hypothalamus is a focus of imprinted gene action**

**Supporting Information**

Matthew J. Higgs^1^, Anna E. Webberley^1^, Alasdair J Allan^2^, Moaz Talat^2^, Rosalind M. John^3^, and Anthony R. Isles^1*^

1 Behavioural Genetics Group, Centre for Neuropsychiatric, Genetics and Genomics, Neuroscience and Mental Health Innovation Institute, Cardiff University, Cardiff, United Kingdom

2 The Mary Lyon Centre, MRC Harwell, Didcot, United Kingdom

3 School of Biosciences, Cardiff University, Cardiff, United Kingdom

* Corresponding author: [islesar1@cardiff.ac.uk](mailto:islesar1@cardiff.ac.uk)

**Supporting Methods**

1. **Preoptic Area (POA) (Moffitt et al., 2018(2))**

The Preoptic area is of particular interest to parenting behaviour and feeding behaviour containing neural populations that contribute to a number of innate hypothalamic behaviour outputs. Moffitt et al. (2018)(2) conducted a molecular, spatial, and functional characterisation of the murine POA and as part of this investigation they profiled 31,299 cells from male and female adult mouse POA. Alongside 21 major cell classes, 18,553 neurons were clustered into 66 distinct neuronal populations (of which 56 originated from the POA). Barcodes, Features and Matrix files were acquired from Gene Expression Omnibus through accession no. GSE113576 and used to create a Seurat object for the analysis. Data were scaled to 10,000 UMI and log10-normalised. Cluster identities were acquired from Supplementary Table 1. accompanying the original publication (<https://science.sciencemag.org/content/362/6416/eaau5324>). Neuronal cluster identities were taken from ‘Neuronal cluster (determined from clustering of inhibitory or excitatory neurons)’ annotations. Data were run through the workflow, once with only POA neurons (removing the 9 suspected extra-POA groups) with upregulated genes capped at 2FC.

1. **Mouse Brain Atlas (Zeisel et al., 2018(3))**

Zeisel et al. (2018)(3) created the Mouse Brain Atlas in 2018 – a comprehensive sequencing of the adolescent mouse nervous system. In our previous analyses, data were downloaded from mousebrain.org (<http://mousebrain.org/downloads.html>) and the Level 5 data were downloaded as a one cell per column loom file which also included the cell metadata as column attributes (“l5.all.loom”). Raw data is also available from SRA repository, [SRP135960](https://www.ncbi.nlm.nih.gov/sra/SRP135960)

1. **Whole Hypothalamus (Chen, Wu, Jiang, and Zhang (2017) (1))**

Chen et al. (2017)(1) sequenced adult (8-10 weeks) whole hypothalamus of female B6D2F1 mice (C57B6 female × DBA2 male). In our previous analyses, processed/normalised data were downloaded as an R object from Gene Expression Omnibus through accession no. [GSE87544](https://www.ncbi.nlm.nih.gov/geo/query/acc.cgi?acc=GSE87544) alongside count data and a metadata file with the cell identities.


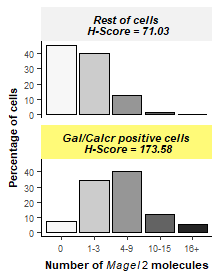

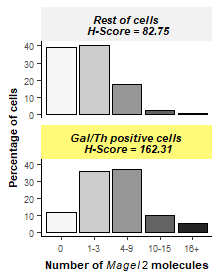


**a**

**b**

**Fig A.** (A) Histogram showing the percentage of cells with particular number of *Magel2* molecules in *Gal/Th* positive cells vs. all other cells. A larger percentage of *Gal/Th* cells expressed 4+ *Magel2* RNA molecules and a smaller percentage of cells expressed zero *Magel2* RNA molecules which contributed to the differences in H-Score. (B) Histogram showing the percentage of cells with particular number of *Magel2* molecules in *Gal/Calcr* positive cells vs. all other cells. A larger percentage of *Gal/Calcr* cells expressed 4+ *Magel2* RNA molecules and a smaller percentage of cells expressed zero *Magel2* RNA molecules which contributed to the differences in H-Score.


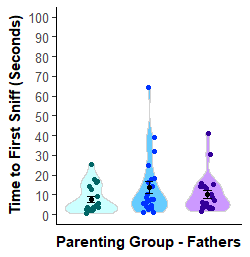

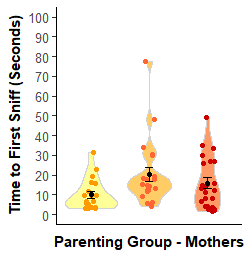

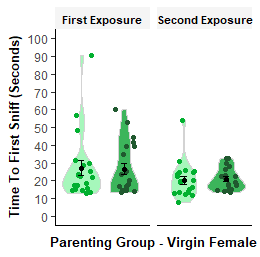

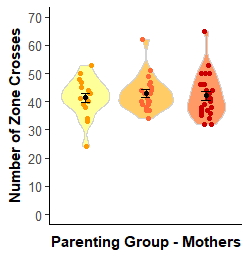

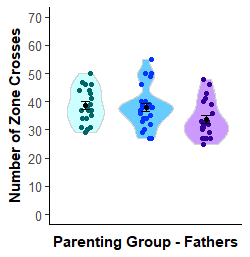

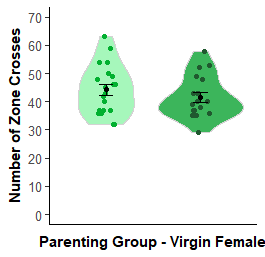

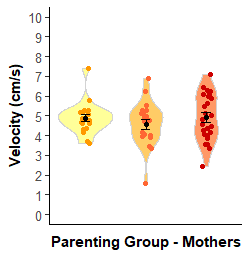

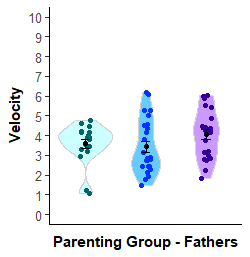

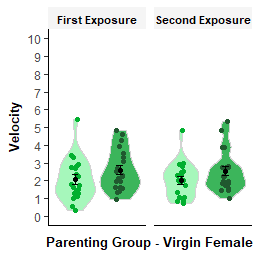

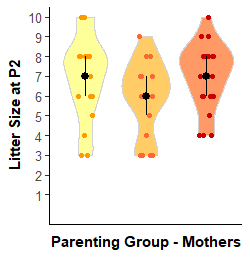


**a**

**b**

**c**

**d**


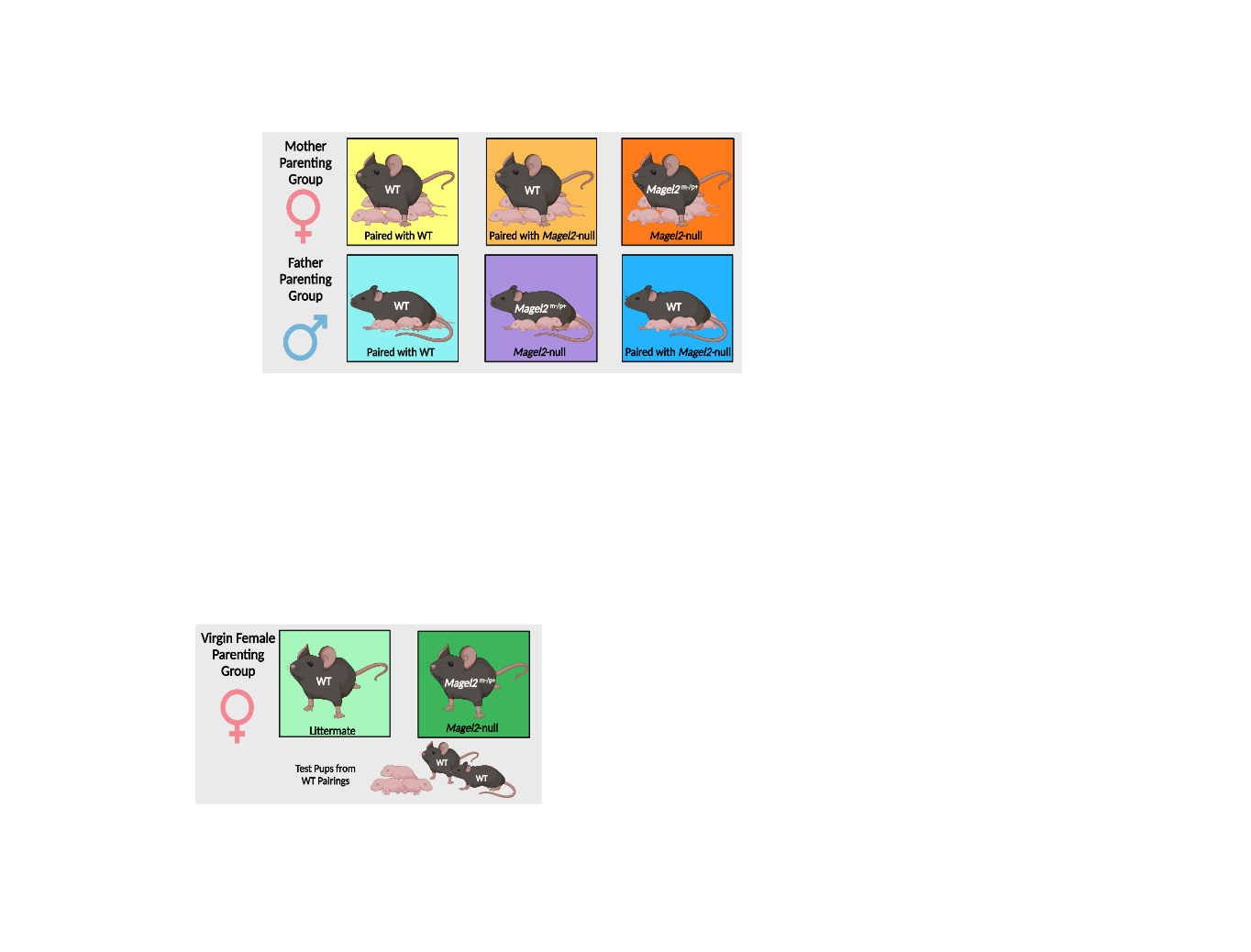

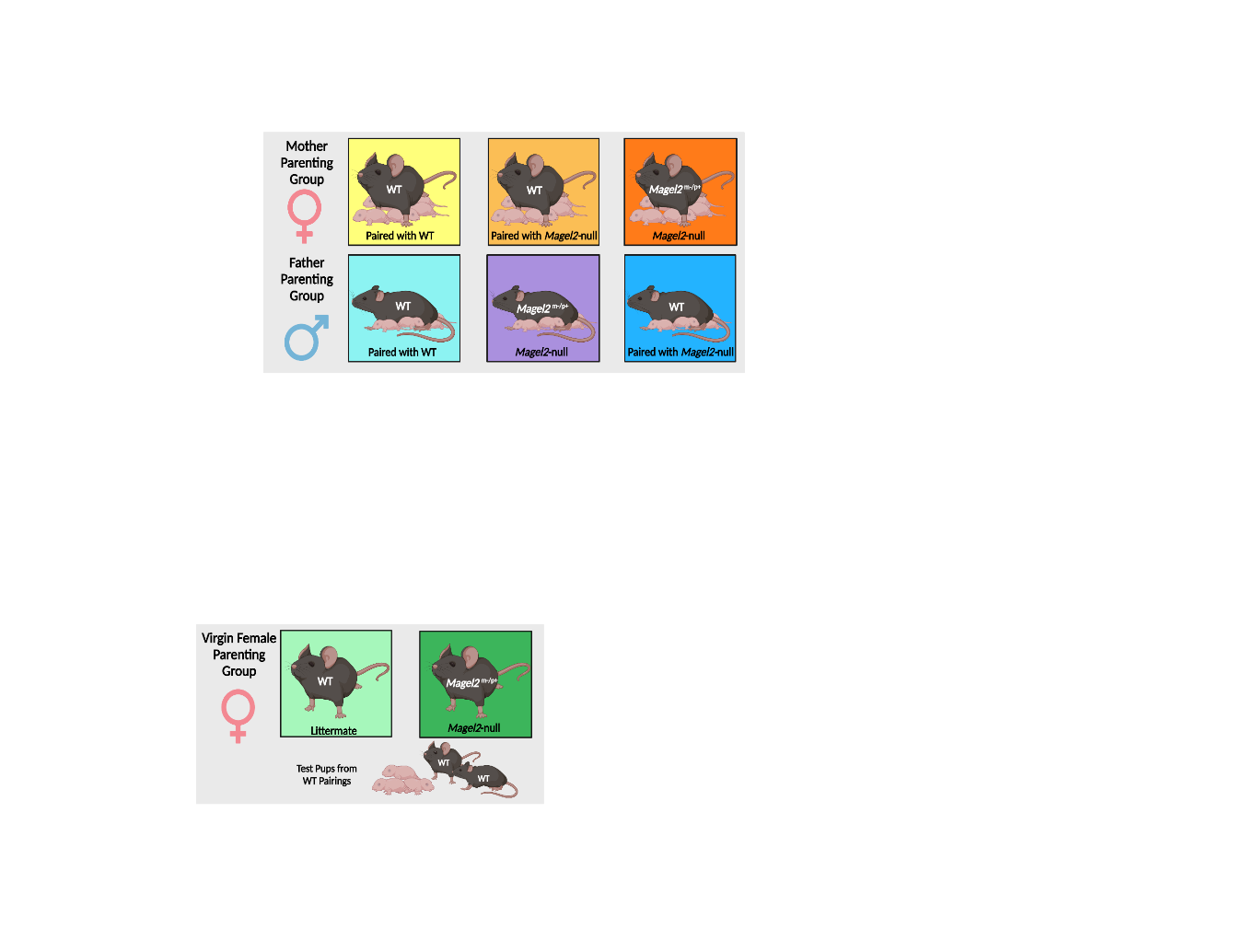


**Fig B. (Previous Page)** Confounders of parenting measured during the retrieval and nest building task and the three chambers task in mothers, fathers and virgin females. (A) Litter size recorded at P2 for the mother/father pairings. (B) Time taken to first sniff a pup in the retrieval and nest building taskfor all three groups of animals. (C) Velocity of each group of animals during the 60-minute retrieval and nest building task measured via automated tracking on Ethovision. (D) The number of chamber crosses in the three-chamber assessment of the three groups of animals measured via automated tracking on Ethovision.

**Fig C** (A) Number of total POA cells per animal used in this study, split by *Magel2*-null and WT (regardless of condition (B) Average number of *c-Fos* molecules present in POA cells of *Magel2*-null mice and WT mice either exposed to pups or controls. (C) Number of *c-Fos* positive (5+ molecules) cells per 1000 Cortical cells (left) and per 1000 POA cells (right) in *Magel2*-null animals vs. WT animals (regardless of exposure). (D) Number of *c-Fos* positive (5+ molecules) cells also expressing *Gal* (2+ molecules) per 1000 POA cells of *Magel2*-null mice and WT mice either exposed to pups or controls. (E) Number of *c-Fos* positive (5+ molecules) cells also expressing *Calcr* (2+ molecules) per 1000 POA cells of *Magel2*-null mice and WT mice either exposed to pups or controls. (F) Average number of *c-Fos* molecules in POA cell also expressing *Gal/Calcr*, *Gal* and *Calcr*, split by *Magel2*-null and WT (regardless of condition).


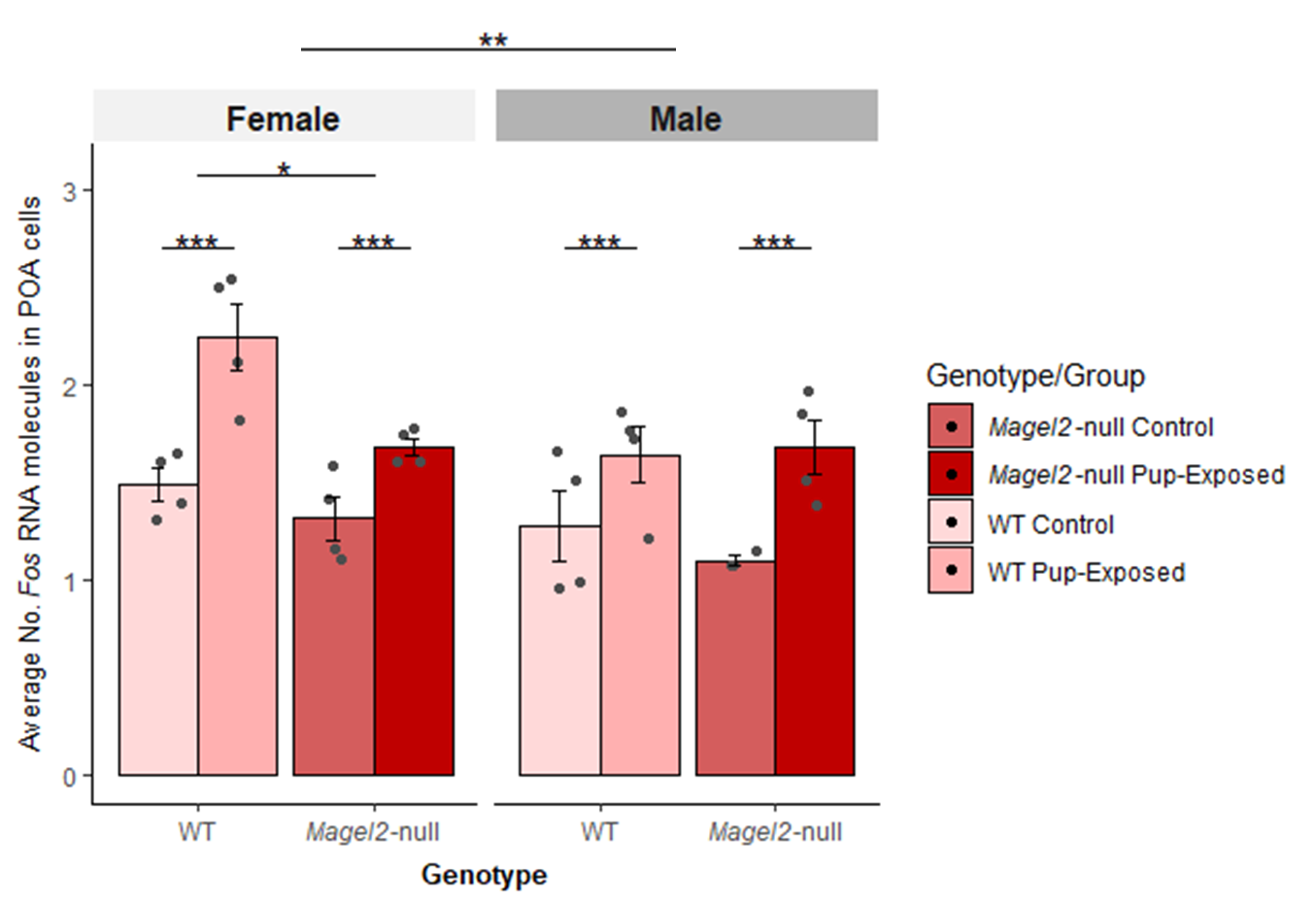

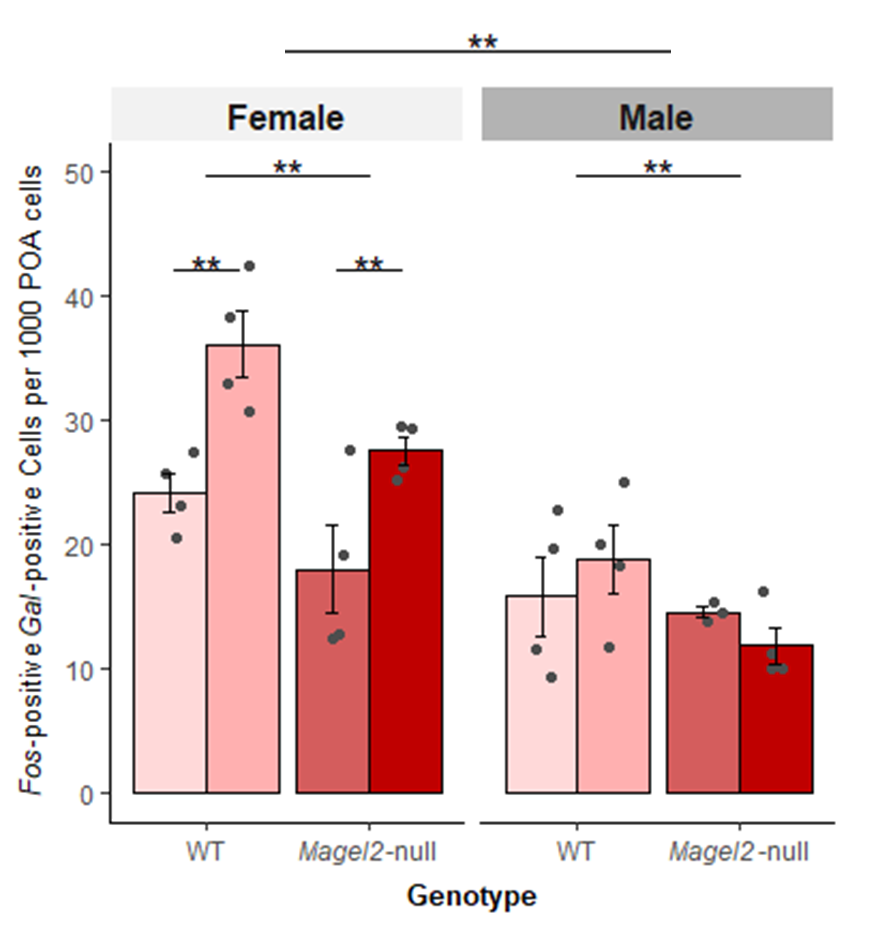

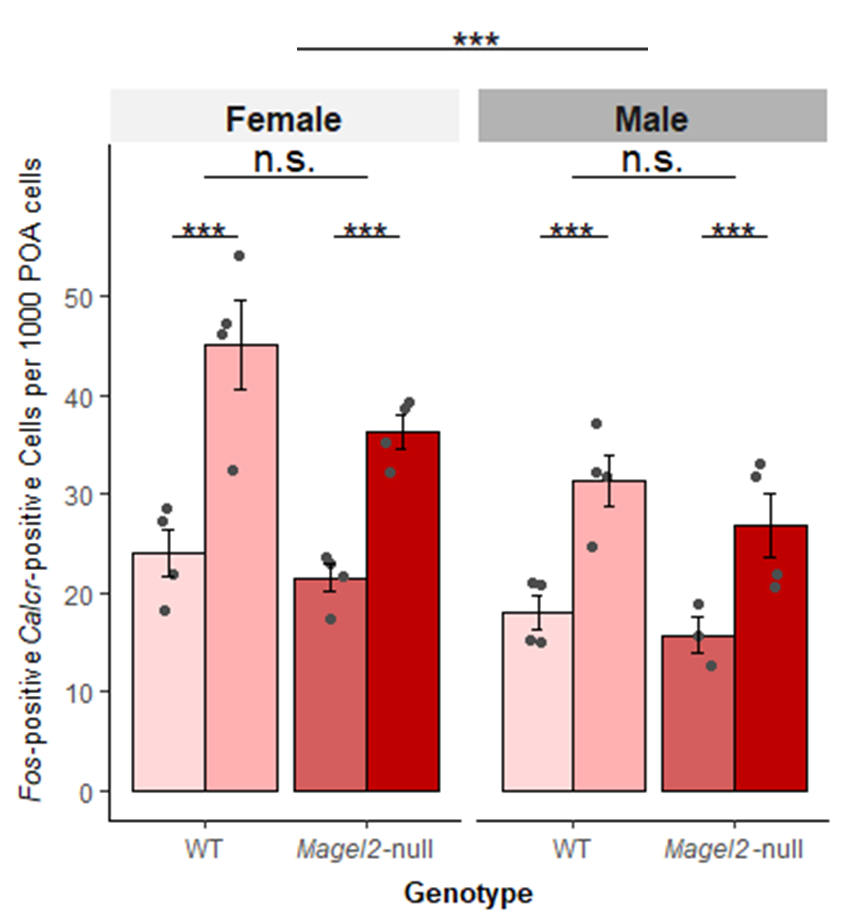

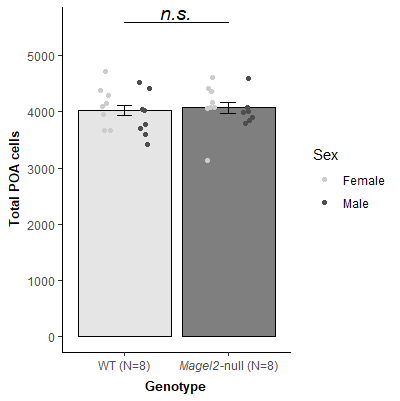

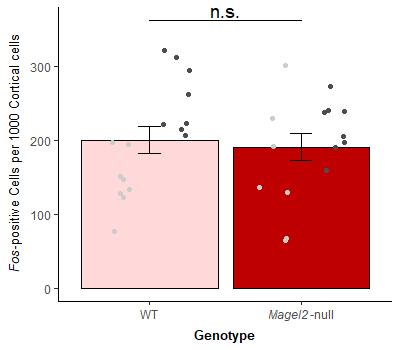

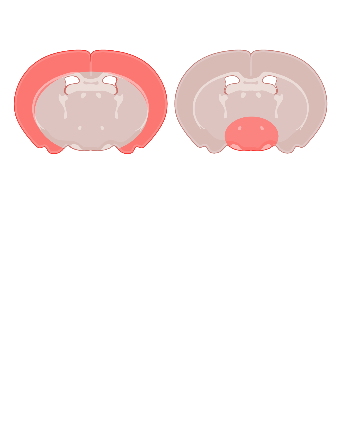

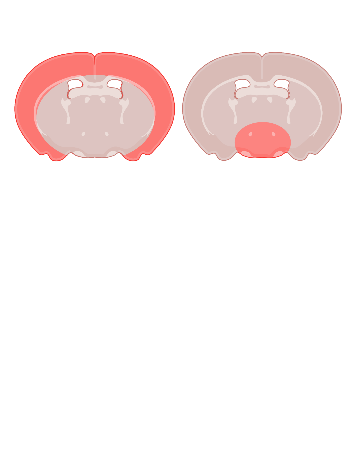

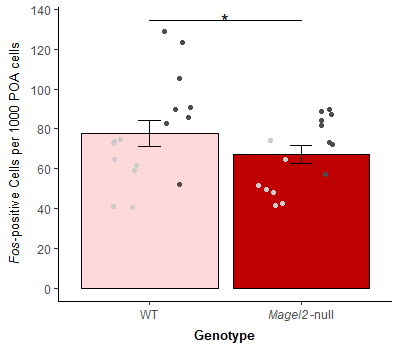

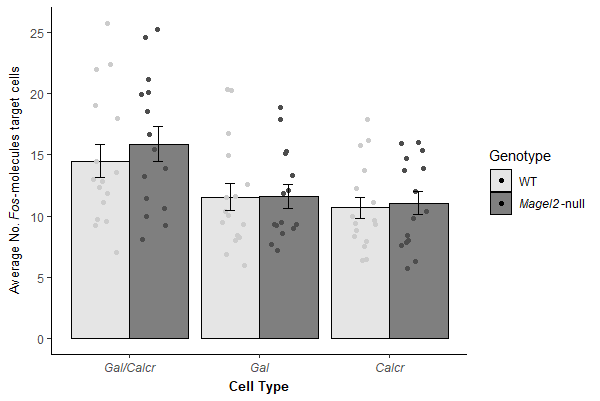

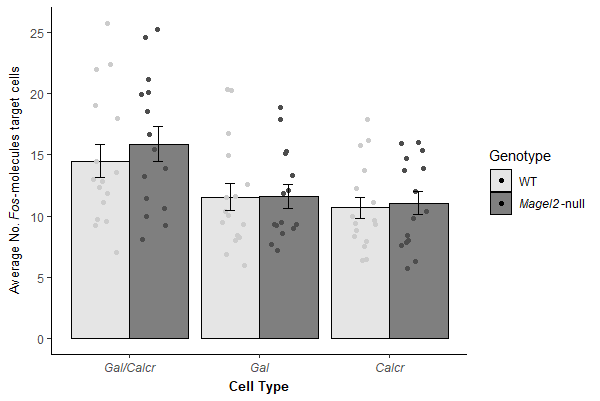


**c**

**d**

**a**

**b**

**e**

**f**


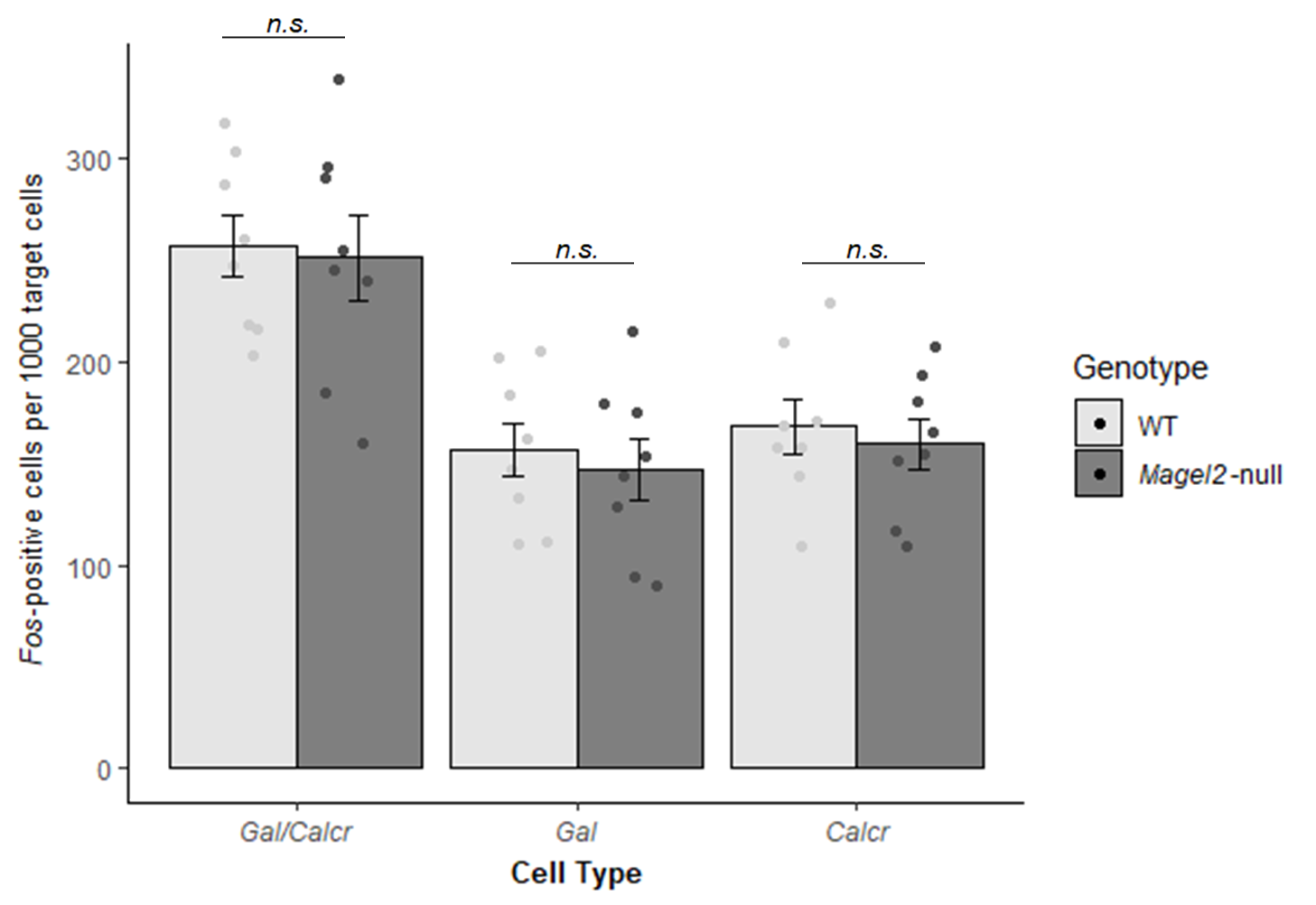

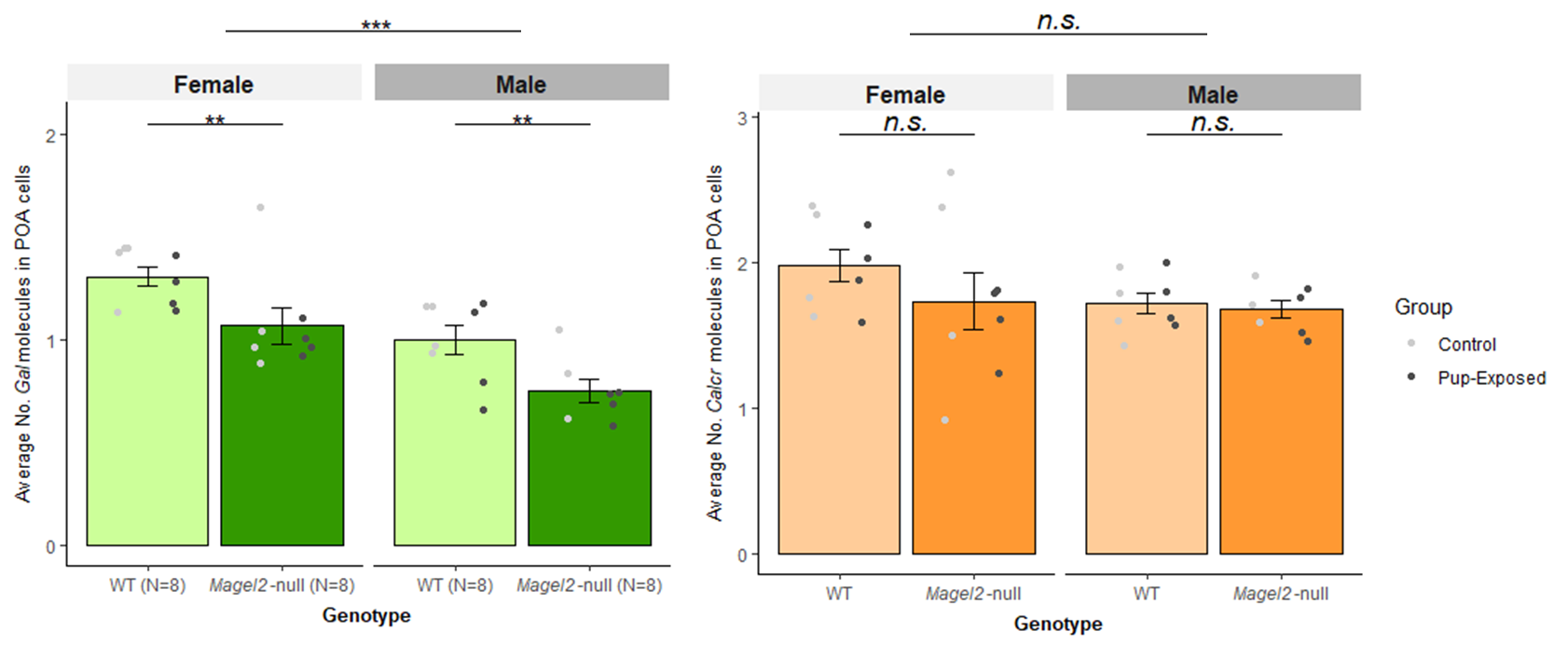


**a**

**b**

**c**

**Fig D.** (A) Average number of *Gal* molecules present in POA cells of *Magel2*-null animals vs. WT animals (regardless of exposure). (B) Average number of *Calcr* molecules present in POA cells of *Magel2*-null animals vs. WT animals (regardless of exposure). (C) Number of (*Gal/Calcr, Gal, Calcr*) cells also registering as *C-Fos* positive (5+ molecules) per 1000 of the respective cell (*Gal/Calcr, Gal, Calcr*). Significant differences seen in Fig 7D & S3D are no longer present here when normalising for *Gal* and *Gal/Calcr* cell number.


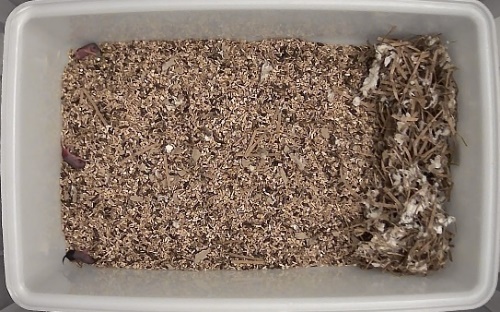

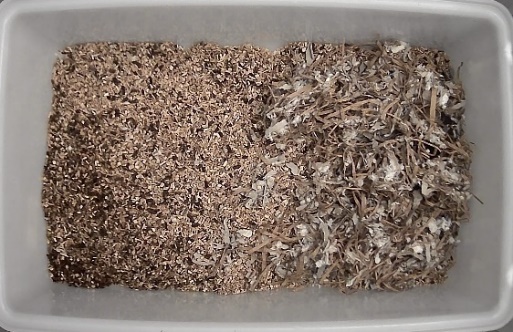


Start
Condition

Level 2 Nest

Level 4 Nest

Level 1 Nest

Level 3 Nest

Level 5 Nest


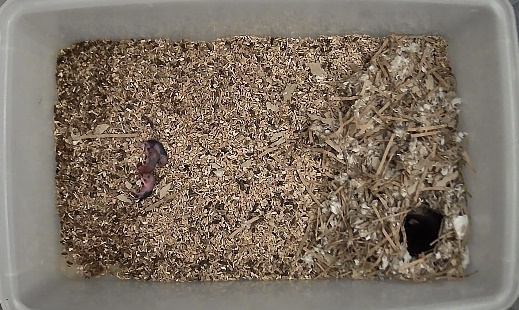

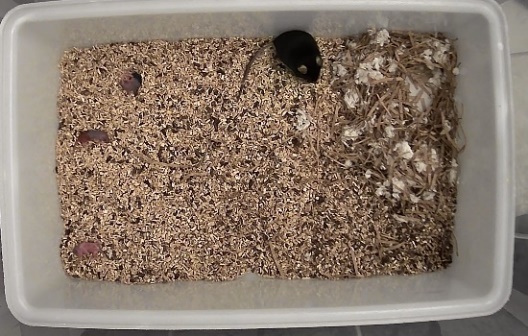


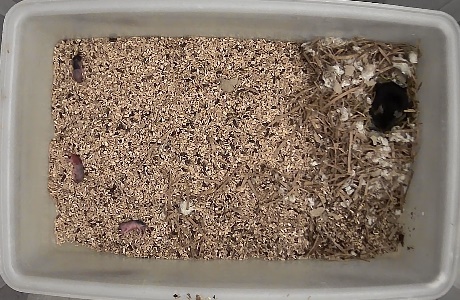

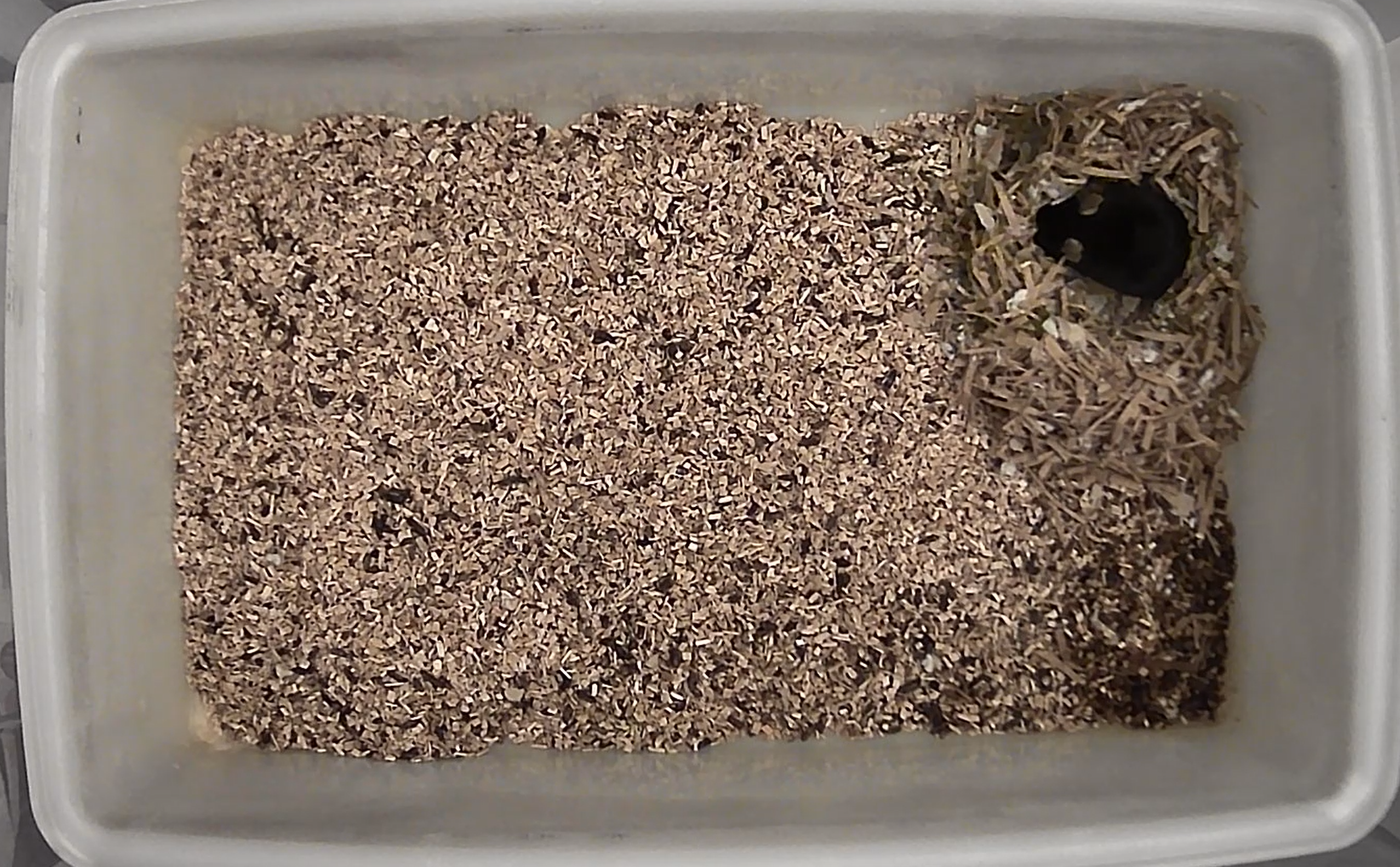


**Fig E.** Exemplar Nest building scoring from the Retrieval-Nest Building assessment. Animals start with nest material fully deconstructed. Level 1 represents no visible nest structure; Level 2 represents an attempt to collect the nest material to one area but no defined nest structure. Level 3 represents a recognisable nest shape using >50% of the material but a large percentage of the material is unused and the structure is not finished. Level 4 represents an almost finished nest >75% of the nest material is used and the nest walls are beginning to form. Level 5 represents a fully formed nest, >90% of the material is used and the nest walls are clearly defined.


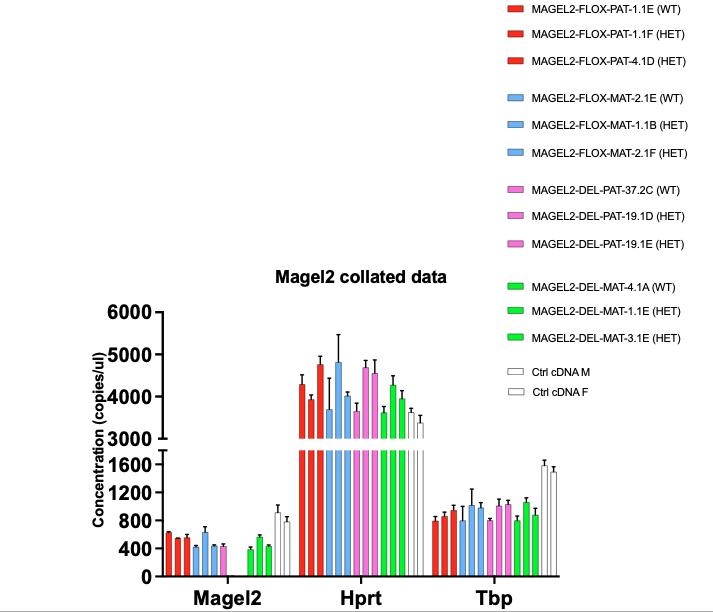


**Fig F.** qRT-PCR on P4 brain from *Magel2*-null mice and controls. Samples tested are detailed in the key but include WT and Het animals following paternal and maternal transmission of a *Magel2* FLOX allele, and WT and Het animals following paternal and maternal transmission of a *Magel2* deleted allele. As is clear from the figure, *Magel2* expression is normal in all samples apart from the Het *Magel2* animals following paternal transmission of the deleted allele. Data for reference genes *Hprt and Tbp* are also provide. Data represent replicate mean +SEM.

| Table A. Specific Hypothalamic Nuclei Level Enrichment Analysis for all imprinted genes in neuronal subpopulation in the Preoptic Area (Moffitt et al., 2018)(2) *Up Reg* – number of upregulated genes with *q* ≤ 0.05 and Log2FC ≥ 1 (total number of genes in the dataset in brackets); *IG* – number of imprinted genes upregulated with *q* ≤ 0.05 and Log2FC ≥ 1 (total number of IGs in the dataset in brackets); *ORA p* – *p* value from over representation analysis on groups with minimum 5% of total IGs; *ORA q* – Bonferroni corrected *p* value from ORA; *Mean FC IG* – mean fold change for upregulated imprinted genes; *Mean FC Rest* – mean fold change for all other upregulated genes;  *GSEA p* – *p* value from Gene Set Enrichment Analysis for identity groups with 15+ IGs and Mean FC IG > Mean FC Rest; *GSEA q* – Bonferroni corrected *p* values from GSEA | | | | | | | | |
| --- | --- | --- | --- | --- | --- | --- | --- | --- |
| POA Neuron Identity | **Up Reg**  **(16,402)** | **IG**  **(100)** | **ORA *p*** | **ORA *q*** | **Mean FC**  **IG** | **Mean FC**  **Rest** | **GSEA *p*** |  |
| i16:*Gal/Th* | 825 | 15 | 0.000137 | **0.002594** | 4.89 | 4.16 | 0.416 |  |
| i35:*Crh/Tac2* | 393 | 9 | 0.000662 | **0.012572** | 2.84 | 5.20 | - |  |
| i8:*Gal/Amigo2* | 353 | 7 | 0.005834 | 0.110848 | 3.81 | 3.67 | - |  |
| e13:*Ghrh/C1ql1* | 1088 | 14 | 0.006165 | 0.117129 | 4.28 | 5.79 | - |  |
| e4:*Trh/Angpt1* | 606 | 9 | 0.011653 | 0.221404 | 3.23 | 5.41 | - |  |
| e15:*Ucn3/Brs3* | 433 | 7 | 0.016667 | 0.316681 | 13.73 | 5.98 | - |  |
| e17:*Th/Adcyap1* | 1659 | 17 | 0.02239 | 0.425404 | 4.39 | 5.21 | - |  |
| i23:*Crh/Nts* | 270 | 5 | 0.024755 | 0.470349 | 2.66 | 4.22 | - |  |
| e22:*Gal/Ucn3* | 1033 | 11 | 0.049722 | 0.944712 | 3.89 | 10.00 | - |  |
| i44:*Th/Cxcl14* | 557 | 7 | 0.053803 | 1 | 8.31 | 6.63 | - |  |
| i37:*Bdnf/Chrm2* | 806 | 9 | 0.057269 | 1 | 5.34 | 5.74 | - |  |
| i22:*Gal/Pmaip1* | 417 | 5 | 0.111348 | 1 | 4.76 | 4.26 | - |  |
| e3:*Cartpt/Isl1* | 799 | 8 | 0.11433 | 1 | 8.73 | 4.94 | - |  |
| e19:*Ghrh/Trh* | 937 | 9 | 0.117593 | 1 | 3.23 | 6.39 | - |  |
| e2:*Tac1/Fezf1* | 569 | 6 | 0.134146 | 1 | 2.77 | 4.13 | - |  |
| i24:*Nmu* | 610 | 6 | 0.168524 | 1 | 2.90 | 4.45 | - |  |
| e10:Glut/*Meis2* | 511 | 5 | 0.201534 | 1 | 3.94 | 5.28 | - |  |
| e16:*Sst/Cartpt* | 1518 | 12 | 0.212905 | 1 | 3.96 | 5.29 | - |  |
| e23:*Reln/Etv1* | 1054 | 8 | 0.313293 | 1 | 3.93 | 8.37 | - |  |
| e1:Glut | 283 | 3 | - | - | 3.39 | 4.22 | - |  |
| e11:Glut/*Shox2* | 676 | 4 | - | - | 4.44 | 7.37 | - |  |
| e12:*Nos1/Foxp2* | 482 | 4 | - | - | 3.77 | 5.42 | - |  |
| e24:*Gal/Rxfp1* | 730 | 4 | - | - | 27.99 | 10.43 | - |  |
| e5:*Adcyap1/Nkx2-1* | 552 | 2 | - | - | 6.55 | 4.10 | - |  |
| e6:*Nos1/Trp73* | 368 | 1 | - | - | 17.60 | 5.84 | - |  |
| e7:*Reln/C1ql1* | 601 | 4 | - | - | 3.40 | 5.18 | - |  |
| e8:*Cck/Ebf3* | 526 | 1 | - | - | 5.84 | 10.53 | - |  |
| e9:Glut/*Tcf7l2* | 459 | 1 | - | - | 4.58 | 5.86 | - |  |
| h1:GABA/*Slc17a6* | 563 | 3 | - | - | 3.30 | 5.99 | - |  |
| h2:*Nts/Slc17a8* | 237 | 1 | - | - | 2.76 | 4.37 | - |  |
| i1:GABA | 74 | 0 | - | - | 0.00 | 3.38 | - |  |
| i10:*Tac1/Nts* | 662 | 3 | - | - | 4.73 | 3.48 | - |  |
| i11:GABA | 172 | 1 | - | - | 5.43 | 3.69 | - |  |
| i12:GABA | 177 | 2 | - | - | 6.02 | 4.01 | - |  |
| i13:GABA | 322 | 2 | - | - | 4.93 | 3.64 | - |  |
| i15:GABA | 412 | 2 | - | - | 3.26 | 4.97 | - |  |
| i17:*Th/Nos1* | 290 | 4 | - | - | 4.71 | 4.41 | - |  |
| i18:*Gal/Tac2* | 516 | 4 | - | - | 2.51 | 3.75 | - |  |
| i2:*Tac1/Pdyn* | 620 | 4 | - | - | 4.65 | 3.63 | - |  |
| i20:*Gal/Moxd1* | 444 | 4 | - | - | 3.47 | 5.26 | - |  |
| i21:*Sst/Pou3f3* | 602 | 4 | - | - | 4.97 | 4.21 | - |  |
| i25:*Npy/Etv1* | 396 | 4 | - | - | 4.19 | 6.07 | - |  |
| i26:*Tac1/Prok2* | 110 | 1 | - | - | 7.04 | 5.15 | - |  |
| i29:GABA*/Igsf1* | 418 | 2 | - | - | 2.52 | 5.21 | - |  |
| i3:*Penk/Nts* | 591 | 3 | - | - | 2.95 | 3.99 | - |  |
| i32:*Sst/Npy* | 350 | 2 | - | - | 3.14 | 7.38 | - |  |
| i38: *Kiss1/Th* | 636 | 3 | - | - | 6.45 | 13.13 | - |  |
| i39:GABA | 331 | 2 | - | - | 2.74 | 7.13 | - |  |
| i4:GABA/*Mylk* | 232 | 2 | - | - | 2.95 | 4.53 | - |  |
| i40:*Sst/Reln* | 270 | 3 | - | - | 7.75 | 7.55 | - |  |
| i41:*Npy/Penk* | 376 | 4 | - | - | 7.15 | 14.72 | - |  |
| i42:*Pthlh* | 375 | 3 | - | - | 5.75 | 8.11 | - |  |
| i43*:Chat* | 1174 | 3 | - | - | 11.91 | 13.93 | - |  |
| i45:*Bdnf/Pmaip1* | 182 | 1 | - | - | 23.06 | 12.12 | - |  |
| i5:GABA/*Pou3f3* | 525 | 1 | - | - | 3.23 | 3.93 | - |  |
| i7:GABA | 261 | 2 | - | - | 4.52 | 4.42 | - |  |
| i9:GABA | 284 | 1 | - | - | 2.09 | 4.12 | - |  |

| Table B. Top 5 neuronal subpopulations showing imprinted gene over-representation in the MBA dataset (Zeisel et al., 2018)(3). *Neuron Description –* more specific classification of neuronal subpopulations as described by original authors; All column descriptions can be found in the legend of S1_Table | | | | | | | | | |
| --- | --- | --- | --- | --- | --- | --- | --- | --- | --- |
| Neuron Identity | **Neuron**  **Description** | **Up Reg**  **(18,335)** | **IG**  **(105)** | **ORA *p*** | **ORA *q*** | **Mean FC**  **IG** | **Mean FC**  **Rest** | **GSEA**  ***p*** | **GSEA**  ***q*** |
| HBSER5 | Serotonergic neurons, hindbrain | 3721 | 53 | 8.29E-12 | **1.19E-09** | 13.76 | 5.98 | 0.002 | **0.042** |
| DEINH5 | Peptidergic neurons, hypothalamus | 611 | 19 | 1.94E-09 | **2.79E-07** | 7.47 | 5.48 | 0.0639 | 1 |
| TEINH3 | Inhibitory neurons, telencephalon | 885 | 22 | 5.50E-09 | **7.93E-07** | 8.60 | 6.38 | 0.2076 | 1 |
| MEINH13 | Inhibitory neurons, midbrain | 1229 | 25 | 2.35E-08 | **3.38E-06** | 9.85 | 7.30 | 0.1213 | 1 |
| HBSER4 | Serotonergic neurons, hindbrain | 3510 | 45 | 2.83E-08 | **4.08E-06** | 27.93 | 6.29 | 5.00E-04 | **0.0105** |

| Table C. Whole Hypothalamus Level Enrichment Analysis for all imprinted genes in neuronal subpopulations in the Chen et al. (2017)(1) dataset. All column descriptions can be found in the legend of S1 Table. Marker Genes – Genes with distinct expression in these neuronal subtypes for the purposes of identification. | | | | | | | |
| --- | --- | --- | --- | --- | --- | --- | --- |
| Hypothalamic Neuron Identity | **Marker**  **Genes** | **Up Reg**  **(12,238)** | **IG**  **(80)** | **ORA *p*** | **ORA *q*** | **Mean FC**  **IG** | **Mean FC**  **Rest** |
| GABA17 | *Slc6a3* | 825 | 18 | 4.57E-06 | **1.19E-04** | 2.73 | 3.30 |
| GABA8 | *Vipr2* | 480 | 11 | 0.0003 | **0.0071** | 1.96 | 2.73 |
| GABA13 | *Slc18a2, Gal* | 569 | 12 | 0.0003 | **0.0079** | 1.89 | 2.02 |
| GABA15 | *Agrp* | 766 | 13 | 0.0013 | **0.0339** | 2.40 | 2.79 |
| GABA16 | *Cox6a2* | 258 | 6 | 0.0068 | 0.1762 | 1.68 | 2.70 |
| GABA14 | *Cbln4* | 271 | 6 | 0.0085 | 0.2220 | 1.68 | 2.54 |
| GABA2 | *Npas1* | 278 | 6 | 0.0096 | 0.2499 | 2.29 | 4.22 |
| GABA18 | *Lhx1, Klhl1* | 200 | 5 | 0.0099 | 0.2576 | 1.55 | 3.06 |
| GABA9 | *Vip* | 219 | 5 | 0.0142 | 0.3703 | 1.39 | 3.77 |
| GABA10 | *Prok2* | 605 | 9 | 0.0169 | 0.4391 | 3.03 | 4.28 |
| Glu9 | *Gng8, Samd3* | 345 | 6 | 0.0252 | 0.6556 | 1.34 | 3.66 |
| Glu1 | *Crh* | 182 | 4 | 0.0312 | 0.8120 | 1.68 | 4.38 |
| GABA11 | *Ghrh* | 392 | 6 | 0.0430 | 1 | 6.50 | 2.91 |
| GABA5 | *Lhx8* | 569 | 7 | 0.0780 | 1 | 1.28 | 3.09 |
| Glu14 | *Avp, Sim1* | 945 | 10 | 0.0875 | 1 | 2.07 | 3.30 |
| Glu15 | *Sst, Prdm8* | 650 | 7 | 0.1319 | 1 | 1.69 | 3.79 |
| Glu12 | *Vgll2* | 339 | 4 | 0.1811 | 1 | 1.70 | 3.88 |
| Glu13 | *Pomc* | 471 | 5 | 0.1945 | 1 | 3.36 | 4.04 |
| GABA12 | *Crabp1* | 354 | 4 | 0.2010 | 1 | 1.63 | 2.61 |
| Glu6 | *Tac1* | 607 | 6 | 0.2052 | 1 | 2.39 | 3.58 |
| Glu7 | *Fezf1, Lbhd2* | 609 | 6 | 0.2072 | 1 | 1.44 | 2.13 |
| Glu11 | *Kiss1* | 468 | 4 | 0.3663 | 1 | 7.78 | 3.68 |
| GABA3 | *Bcl11b* | 484 | 4 | 0.3902 | 1 | 1.41 | 3.36 |
| Glu8 | *Lbhd2, Cartpt* | 534 | 4 | 0.4641 | 1 | 3.34 | 2.97 |
| Glu4 | *Shox2* | 1690 | 8 | 0.8792 | 1 | 2.25 | 3.09 |
| Glu5 | *Foxb1* | 984 | 4 | 0.8942 | 1 | 2.20 | 3.69 |
| GABA6 | *Pax6* | 228 | 3 | - | - | 1.03 | 3.86 |
| GABA7 | *Trh* | 194 | 3 | - | - | 3.87 | 3.46 |
| Glu3 | *Fezf2, Samd3* | 203 | 3 | - | - | 1.07 | 5.35 |
| GABA1 | *Pvalb* | 738 | 2 | - | - | 3.81 | 4.57 |
| GABA4 | *Gm13498* | 254 | 2 | - | - | 3.97 | 4.43 |
| Glu10 | *Trh* | 228 | 2 | - | - | 1.17 | 4.88 |
| Hista | *Hdc* | 585 | 2 | - | - | 3.98 | 5.14 |
| Glu2 | *Sln* | 376 | 0 | - | - | 0.00 | 4.38 |

| **Table D.** Outcome of statistical tests for *Gal/Th* and *Gal/Calcr* *Magel2* expression comparisons. For the *Gal/Th* (top) and *Gal/Calcr* (bottom) experiments, below are the results of comparing the number of *Magel2* molecules present in these cells to the rest of all POA cells, the other *Gal* positive POA cells as well as the *Th/Calcr* positive cells. Finally, a comparison was made when removing all Magel2 non-expressing cells from the analysis and so assessing whether *Magel2* was more strongly expressed in target cells vs. other *Magel2* expressing cells. | | | | | | |
| --- | --- | --- | --- | --- | --- | --- |
| **Group (N)** | **No. of Cells** | **% Cells *Magel2* Positive** | **Avg. No. *Magel2* molecules** | **Statistical Test** | **Test statistic vs. target group** | ***p* value vs. target group** |
| ***Gal/Th* POA (N=2) compared to ...** | 2579 | 88.29% | 5.28 |  |  |  |
| **Rest of All POA Cells (N=2)** | 66,981 | 61.26% | 2.11 | Mann Whitney U | W = 47192483 | < 2.2x10-16 |
| **Rest of POA *Gal* (N=2)** | 2,511 | 82.99% | 4.11 | Bonferroni Corrected Dunn Test | Z = 6.87 | 3.85x10-11 |
| **Rest of POA *Th* (N=2)** | 12,912 | 77.17% | 3.22 | Bonferroni Corrected Dunn Test | Z = 19.91 | 2.14x10-87 |
| **Rest of POA Cells (No *Gal*/No *Th*) (N=2)** | 51,558 | 56.22% | 1.74 | Bonferroni Corrected Dunn Test | Z = 46.5 | 0.00 |
| ***Gal/Th* *Magel2* +ve POA (N=2) compared to …** | 2,277 | 100.00% | 5.99 |  |  |  |
| **Rest of All POA Cells *Magel2* +ve (N=2)** | 41,033 | 100.00% | 3.45 | Mann Whitney U | W = 30882369 | < 2.2x10-16 |
| ***Gal/Calcr* POA (N=4) compared to …** | 3846 | 92.23% | 5.81 |  |  |  |
| **Rest of All POA Cells (N=4)** | 152,323 | 54.48% | 1.59 | Mann Whitney U | W = 110992285 | < 2.2x10-16 |
| **Rest of POA *Gal* (N=4)** | 23,512 | 76.72% | 3.06 | Bonferroni Corrected Dunn Test | Z = 32.64 | 7.34x10-233 |
| **Rest of POA *Calcr* (N=4)** | 4,818 | 84.81% | 3.30 | Bonferroni Corrected Dunn Test | Z = 19.08 | 3.46x10-81 |
| **Rest of POA *Cells* (No *Gal*/No *Calcr*) (N=4)** | 123,993 | 49.09% | 1.24 | Bonferroni Corrected Dunn Test | Z = 77.54 | 0.00 |
| ***Gal/Calcr* Magel2 +ve POA (N=4) compared to …** | 3,547 | 100% | 6.30 |  |  |  |
| **Rest of All POA Cells *Magel2* +ve (N=4)** | 82,990 | 100% | 2.91 | Mann Whitney U | W = 75812992 | < 2.2x10-16 |

| **Table E.** Proportion of different cell types with varying numbers of *Magel2* reads and the corresponding H-scores. H-scores are calculated from weighted calculation of proportions using the guidance from ACD Bio-techne (SOP 45-006) | | | | | | | |
| --- | --- | --- | --- | --- | --- | --- | --- |
| ***Cell Type*** | **0 *Magel2* reads** | ***1-3 Magel2* reads** | **4-9 *Magel2* reads** | **10-15 *Magel2* reads** | **16+ *Magel2* reads** | **H-Score** | **H-score - only expressors** |
| **POA - *Gal/Calcr*** | | | | | | | |
| ***Gal/Calcr*** | 0.08 | 0.37 | 0.43 | 0.13 | 0.06 | 173.583 | 188.215 |
| ***Gal*** | 0.30 | 0.59 | 0.33 | 0.06 | 0.02 | 115.379 | 150.385 |
| ***Calcr*** | 0.18 | 0.60 | 0.34 | 0.05 | 0.01 | 125.633 | 148.140 |
| **Rest** | 0.84 | 0.74 | 0.23 | 0.03 | 0.01 | 71.029 | 130.370 |
| **POA - *Gal/Th*** | | | | | | | |
| ***Gal/Th*** | 0.13 | 0.40 | 0.42 | 0.12 | 0.06 | 162.311 | 183.838 |
| ***Gal*** | 0.20 | 0.47 | 0.40 | 0.10 | 0.03 | 85.941 | 168.762 |
| ***Th*** | 0.30 | 0.58 | 0.34 | 0.06 | 0.02 | 140.064 | 152.579 |
| **Rest** | 0.63 | 0.66 | 0.29 | 0.04 | 0.01 | 117.743 | 140.287 |

| Table F. Statistical results from mother and father analyses carried out without including parents with test litters containing mutant pups. Statistical tests used are the same as reported in the main text P values in red indicate disparities with the original statistical analysis. | | | | | |
| --- | --- | --- | --- | --- | --- |
| Mothers (N=11) | | | **Pairwise p** | | |
| Measure | **Test Stat** | **p** | **WT(WT) vs. WT(HET)** | **WT(WT) vs. HET** | **WT(HET) vs. HET** |
| Task Fin | 20.72 | **2.59x10^-7^** | 1 | **6.7x10^-7^** | **0.00021** |
| Task Status | 15.727 | **0.00038** | 1 | **0.001** | **0.01** |
| No. Pups Ret | NA | NA | NA | NA | NA |
| First Ret | 1.34 | 0.271 | NA | NA | NA |
| Last Ret | 2.804 | 0.069 | NA | NA | NA |
| Level 3 Nest | 20.72 | **2.59x10^-7^** | 1 | **6.7x10^-7^** | **0.00021** |
| Nest Quality | 17.363 | **0.00017** | 1 | **0.0003** | **0.015** |
| PDB Ret | 6.862 | **0.002** | 1 | **0.0043** | **0.035** |
| PDB Fin | 19.26 | **5.88x10^-7^** | 1 | **1.8x10^-6^** | **0.00024** |
| Fathers (N=11) | | | **Pairwise p** | | |
| Measure | **Test Stat** | **p** | **WT(WT) vs. WT(HET)** | **WT(WT) vs. HET** | **WT(HET) vs. HET** |
| Task Fin | 14.771 | **0.0006** | 0.676 | **0.0004** | **0.0093** |
| Task Status | 22.391 | **1.374 x 10^-5^** | 0.88 | **1.4x10^-5^** | **0.0003** |
| No. Pups Ret | 18.315 | **0.0001** | 1 | **0.0002** | **0.00038** |
| First Ret | 6.53 | **0.003** | 1 | **0.0026** | **0.0159** |
| Last Ret | 15.833 | **0.0004** | 1 | **0.0005** | **0.001** |
| Level 3 Nest | 16.512 | **0.0003** | 0.19 | **0.0001** | **0.02** |
| Nest Quality | 11.327 | **0.003** | **0.044** | **0.004** | 0.61 |
| PDB Ret | 4.597 | **0.0145** | 1 | **0.014** | **0.052** |
| PDB Fin | 6.29 | **0.0036** | 0.466 | **0.0025** | **0.047** |

| Table G. Intra class correlation coefficient (ICC) Kappa and *p* values as measures of the primary and second scorer inter-rater reliability. Statistics were calculated from the 80/210 videos that were second scored | | |
| --- | --- | --- |
| Metric from Retrieval/Nest Building Assessment | **ICC Kappa (0-1)** | **ICC *p* value** |
| *Time to Retrieve Pup 1* | 0.915 | 1.89x10^-33^ |
| *Time to Retrieve Pup 2* | 0.936 | 3.05x10^-38^ |
| *Time to Retrieve Pup 3* | 0.958 | 3.05x10^-45^ |
| *Number of Pups Retrieved* | 0.935 | 6.12x10^-38^ |
| *Time to Build a Level 3 Nest* | 0.894 | 7.44x10^-30^ |
| *Final Nest Quality Rating* | 0.778 | 4.61x10^-18^ |
| *Time Until Task Finished* | 0.872 | 9.33x10^-27^ |
| *PDB Until Retrieval* | 0.853 | 2.36x10^-24^ |
| *PDB Until Task Finished* | 0.928 | 3.46x10^-36^ |

| **Table H.** Settings used for each gene/probe combination when acquiring images used Zeiss AxioScan Z1. Gene/probe combinations are listed with the catalogue code the probe was registered under in the ACD Biotechne catalogue and the specific experiment the probe was used in within this thesis. For each probe combination, the intensity percentage and light duration (milliseconds (ms)) are recorded. | | | | |
| --- | --- | --- | --- | --- |
| **Experiment** | **Gene / Probe** | **Probe Catalogue code** | **HXP**  **120 V Intensity** | **HXP**  **120 V Duration** |
| **TSA Vivid**^TM^ **520 (Green, Fluorescein)** | | | | |
| *Calcr/Gal/Magel2* | *Calcr* | ACD 494071,Mm-Calcr | 50% | 1500ms |
| *Gal/Calcr/Fos & Gal/Th/Magel2* | *Gal* | ACD 400961, Mm-*Gal* | 50% | 400ms |
| *Oxt/Avp/Magel2* | *Oxt* | ACD 493171 Mm-*Oxt* | 50% | 100ms |
| **TSA Vivid**^TM^ **570 (Orange, Cy3)** | | | | |
| *Calcr/Gal/Magel2* | *Gal* | ACD 400961-C2, Mm-*Gal*-C2 | 100% | 15ms |
| *Gal/Th/Magel2* | *Th* | ACD 317621-C2, Mm-*Th*-C2 | 100% | 25ms |
| *Gal/Calcr/Fos* | *Calcr* | ACD 494071-C2,Mm-Calcr-C2 | 100% | 10ms |
| *Oxt/Avp/Magel2* | *Avp* | ACD 401391-C2, Mm-*Avp*-C2 | 100% | 2.5ms |
| **TSA Vivid**^TM^ **650 (Red, Cy5.5)** | | | | |
| *Calcr/Gal/Magel2, Gal/Th/Magel2, Oxt/Avp/Magel2* | *Magel2* | ACD 502971-C3, Mm-*Magel2*-C3 | 100% | 150ms |
| *Gal/Calcr/Fos* | *Fos* | ACD 316921-C3, Mm-*Fos*-C3 | 100% | 200ms |

**References**

1. Chen R, Wu X, Jiang L, Zhang Y. 2017. Single-cell RNA-seq reveals hypothalamic cell diversity. *Cell reports* **18**: 3227-3241.
2. Moffitt JR, Bambah-Mukku D, Eichhorn SW, Vaughn E, Shekhar K, Perez JD, Rubinstein ND, Hao J, Regev A, Dulac C. 2018. Molecular, spatial, and functional single-cell profiling of the hypothalamic preoptic region. *Science* **362**: eaau5324.
3. Zeisel A, Hochgerner H, Lönnerberg P, Johnsson A, Memic F, Van Der Zwan J, Häring M, Braun E, Borm LE, La Manno G. 2018. Molecular architecture of the mouse nervous system. *Cell* **174**: 999-1014. e1022.
