# Supplementary material for: The parenting hub of the hypothalamus is a focus of imprinted gene action
Source: PLoS Genet. 2023 Oct 19;19(10):e1010961. doi: 10.1371/journal.pgen.1010961 (PMC10586610; doi:10.1371/journal.pgen.1010961)
Supplement: Attachment — Submitted filename: Review F. Muscatelli.docx [file pgen.1010961.s005.docx]

This paper reveals the impact of imprinted genes in parenting based on the expression level of these genes in galanin-expressing POA neurons, known to be involved in parenting. The study then focused on the imprinted gene Magel2, confirming the high level of expression in galanin neurons and investigating the role of Magel2 in parental behaviour. Magel2 KO mothers, fathers and virgin females showed alterations in parental behaviour. Magel2 KO females show a reduction in the number of galanin neurons and a reduction in the number of these cFos positive cells after pup-exposure , suggesting a decrease in the neuronal activity of galanin neurons.

This is an interesting article, very well written. The genetic control of parental behaviour, both maternal and paternal, is a very interesting and yet little studied topic. This article reveals a particular role of imprinted genes in this behaviour and thus proposes a new functional role of this gene family in the parent-child relationship. The research is well conducted using single cell RNA-seq analysis, RNAscope studies and behavioural studies to assess parental behaviour. The results are clear and the data analyses are robust.

Major comments:

-The Magel2 null mouse line is identified as "Magel2-FLOX-EM1.1". It is necessary to describe this mouse line if it is a novel line or to give a reference (publication) where the mouse line is described.

-The paragraph concerning cFos activity (line 348 to 382) is written taking into account, on the one hand, the genotype and, on the other hand, the sex. As it stands, it is difficult to read the comments and look at the figures. This paragraph should be written taking into account sex and genotype by comparing mutant males with the control and mutant females with the control (as in the figures), it will be easier to understand.

Minor comments :

-Figure S3b (B): There is no difference in WT exposed-pups males compared to WT controls but in Magel2-null there is a difference. Why is this?

-Additional data format: citations in the text of table S2A and B disrupt the text.

-It would have been interesting to have, as in females, the data on cFos expression in Galanine neurons in Magel2KO fathers and virgin females compared to WT fathers.
