# Supplementary material for: The parenting hub of the hypothalamus is a focus of imprinted gene action
Source: PLoS Genet. 2023 Oct 19;19(10):e1010961. doi: 10.1371/journal.pgen.1010961 (PMC10586610; doi:10.1371/journal.pgen.1010961)
Supplement: Attachment — Submitted filename: Higgs et al revision_Cover letter.docx [file pgen.1010961.s006.docx]

Professors Bartolomei and Bickmore

Editorial Board

*PLoS Genetics*

26 July 2023

Dear Marissa and Wendy

**RE: Revision of PGENETICS-D23-00392**

On behalf of my co-authors, I am submitting a revised version of our manuscript entitled “The parenting hub of the hypothalamus is a focus of imprinted gene action” (PGENETICS-D-23-00392) to be considered for publication in *PLoS Genetics*.

As indicated in your decision letter the reviewers described the research as “well-conceived” and an “elegant study”, which was “very well written”. They also highlight that the “genetic control of parental behaviour, both maternal and paternal, is a very interesting yet little studied topic.”

However, all reviewers raised a number of minor issues. In response to this constructive criticism, we have revised the manuscript and highlighted the text changes throughout. A point-by-point response (in *italics*) to each is detailed below.

A version of the manuscript has been uploaded to bioRxiv (<https://doi.org/10.1101/2023.03.27.534088>). If accepted, we will revise the bioRxiv submission, and upload the final accepted version of the manuscript.

The authors state they have no conflicts of interest.

We feel the reviewers suggestions and our revisions have improved the manuscript. We feel these studies, in addition to being highly relevant for the genomic imprinting community, will also be of relevance to the wider *PLoS Genetics* readership, particularly those with an interest in neuroscience and evolution. We hope you agree, and that the manuscript is now acceptable for publication in *PLoS Genetics*.

Sincerely

**Anthony Isles**

Professor of Molecular and Behavioural Neuroscience

Telephone | Ffôn: +44 (0)29 2068 8467\

E-mail | E-bost: [IslesAR1@cardiff.ac.uk](mailto:IslesAR1@cardiff.ac.uk)

**RESPONSE TO REVIEWERS**

**Reviewer #1:**

“The Magel2 null mouse line is identified as "Magel2-FLOX-EM1.1". It is necessary to describe this mouse line if it is a novel line or to give a reference (publication) where the mouse line is described”

*We have added further detail to the description of the line in the METHODS section, including adding gene expression data showing that Magel2 expression is lost when the deletion is paternally transmitted only. As a consequence of including these data, we have also added two new authors to the manuscript. The additional text is below:*

*“….Magel2-null mice (paternal transmission of ablated allele) and their WT littermates on a C57BL/6J background. Magel2-null mice carried a constitutive deletion derived from a Magel2-FLOX-EM1.1 line generated by the Mary Lyon Centre (MLC) at MRC Harwell, Oxford UK. Genomic QC on this line confirmed correct deletion within the single exon and loss of expression in P4 brain was confirmed by qPCR (Supplemental Figure S6).”*

The paragraph concerning c-Fos activity (line 348 to 382) is written taking into account, on the one hand, the genotype and, on the other hand, the sex. As it stands, it is difficult to read the comments and look at the figures. This paragraph should be written taking into account sex and genotype by comparing mutant males with the control and mutant females with the control (as in the figures), it will be easier to understand.

*Although there are differences in c-Fos activity between males and dams, there was no statistical interaction, and the direction and magnitude of change in Magel2-null mice was similar for both males and females. Therefore, we feel it is less confusing, and possibly a truer reflection of the data, to discuss the differences between mutants and WT without breaking them down by sex. However, to address the difficulty in understanding this paragraph, we have added several sentences to this paragraph to indicate that both sexes are being described and that they are showing the same direction of effect. We believe this increases the comprehension of the paragraph without increasing the paragraph to excessive lengths.*

Figure S3b (B): There is no difference in WT exposed-pups males compared to WT controls but in Magel2-null there is a difference. Why is this?

*We thank the reviewer for highlighting this odd result. We had originally assumed this may be due a stochastic issue with RNA molecule expression. However, digging into the data, we realised that the average values for the number of molecules (data presented in Figure S3B, S4A and S4B) were not calculated correctly, the data had been summed across sections rather than averaging between them. With the correct values, this lack of difference between WT pup-exposed and controls no longer exists and the number of c-Fos molecules in Figure S3B now mirrors the results seen for number of c-Fos positive cells in Figure 7B. Figures S3B. S4A and S4B have been updated with the correct values for the average number of molecules per cell. Critically, the statistical tests for these data had been carried out correctly and the error only manifested in the code to produce the figures.*

Additional data format: citations in the text of table S2A and B disrupt the text.

*We have now removed several citations from the text of table S2A and S2B which now read as follows:*

- ‘Supplemental Table S2A. Top 5 neuronal subpopulations showing imprinted gene over-representation in the MBA dataset (Zeisel et al., 2018). *Neuron Description –* more specific classification of neuronal subpopulations as described by original authors; All column descriptions can be found in the legend of Supplemental Table S1’
- ‘Supplemental Table S2B. Whole Hypothalamus Level Enrichment Analysis for all imprinted genes in neuronal subpopulations in the Chen et al. (2017) dataset. All column descriptions can be found in the legend of Supplemental Table S1. Marker Genes – Genes with distinct expression in these neuronal subtypes for the purposes of identification.’

It would have been interesting to have, as in females, the data on c-Fos expression in Galanin neurons in Magel2KO fathers and virgin females compared to WT fathers.

*We agree with the reviewer that these comparisons would be interesting. Figure S3D displays the RNAscope c-Fos data for Magel2KO and wild-type fathers in galanin neurons which shows a similar loss of c-Fos activity in the male Magel2KO. However, there was far fewer c-Fos active Galanin neurons in male POA compared to the dams. For virgin females, we did not perform an RNAscope experiment as we did not expect any particular further insight into activation over and above that seen the males as these show very similar levels of behaviour to virgin females.*

**Reviewer #2:**

The experiment with virgin females indicates that they may have improved their ability to perform the task during the second exposure, suggesting that the pup retrieval test may not necessarily reveal a deficiency in parenting behaviour. Instead, the mutant mice (mothers, fathers, and virgin females) may simply be slower to respond to unfamiliar situations created by the experimental conditions. Are there any differences in "normal" parenting observed in the home cage? Additionally, considering certain neural disorders like autism that do not necessarily result in poor parenting but can affect an individual's ability to cope with new and unfamiliar situations, do the authors believe that a naturalistic approach to assessing parental behaviour would produce different results?

*We agree with the reviewer that a naturalistic assessment of parenting behaviour would be a fascinating next step. We did not formally assess naturalistic parenting behaviour in the home cage here, however from general observations, there were no substantial parenting differences in the home cage i.e., nest quality, times found nursing, neonatal lethality. It is important to note also that the required breeding design would possibly hamper any true test of naturalistic parenting because every mutant animal was cohoused with a WT partner, meaning any deficits could effectively be masked by the WT partner “picking up the slack”. Nevertheless, our prediction is that a naturalistic assessment with two mutant animals would produce similar results, poorer quality nests and less time spent in pup-directed behaviour.*

*Regarding whether our parenting deficits could be the result of an individual’s ability to cope with new and unfamiliar situations, this may be a possibility, as it could be for any behavioural test. As outlined in the materials and methods (Lines 524-525 & 553-556) animals we habituated to the test situation in order minimize the impact of the novel environment (also see response to Reviewer 2, comment 3). Moreover, one might expect an inability to adapt to novelty would also manifest in other behavioural features, such as time to first sniff and interact with pups, velocity and general movement patterns etc. We found no differences between mutant and WT animals on these measures, which suggest the mutant animals engaged with the task in a very similar manner to WT animals, but they were just less successful at the specific parenting aspects.*

*To address this directly, we have added a sentence addressing these concerns about the origin of the behavioural deficits in our mice*

- *Line 173-175. “Finally, to reduce the novelty aspect of these tests, the test was performed in the home cage with the home nest and their own pups, and animals were thoroughly habituated to the apparatus in the preceding days.”*

In a study by Bosque Ortiz and colleagues (<https://doi.org/10.1111/gbb.12776>), it was reported that loss of Magel2 impairs separation-induced vocalization of the offspring and maternal behaviour towards these pups, which appears to be in contrast with the findings by Higgs. Can the authors comment on this? One possible explanation is that the time window considered by Higgs (P3-P5) precedes the one examined in the Bosque Ortiz paper.

*We agree with the reviewer that the difference in time window is the most likely explanation for why we saw no effect of pup vocalisation on retrieval. It is also important to note that Bosque Ortiz et al. saw an effect of loss of Magel2 on maternal preference for retrieval (i.e., when given a clear choice, they retrieve WT pups first before mutant pups). Whether this effect would also be observed in our mixed pup genotype retrieval set-up, as all our test litters had at one or more WT pups, it would be difficult to confirm whether USV production of the mutants would have an influence. We have included a sentence in the discussion to draw attention to this.*

- *Line 417-419 - “Here we have shown that loss of Magel2 results in deficits in parenting behaviour independent of pup USV production. USVs have been shown to affect maternal care at later developmental stages (36) but at the time window of this study, they appear to not have an impact on this retrieval paradigm.”*

The nest building and pup retrieval test is known to induce stress in mice. Given this, I am curious about how Magel2-mutant mice cope with stress and whether this plays a role in their performance. While the test itself is inherently stressful and difficult to limit in a traditional setting, I am wondering if the authors took any measures to reduce the stress effects.

*We were aware that these tests were potentially stressful and put measures in place to minimise these effects. All mice were tube-handled and were paired and tested by the same individual (MJH). Retrieval/Nest building assessments were performed in the home cage, with home nest material and their own pups. All mice received habituation to the test room for 3 days prior to the test assessment. This involved 20 minutes of exposure each day to the set up and 20 minutes prior to the test beginning. The habituation and measures to reduce stress are described in detail in Lines 524-525 & 553-556*

The authors stated that the pup retrieval-nest building test was considered finished when all the pups were retrieved, and the nest was reconstructed to achieve a level 3 quality. If possible, I suggest they include a scale with relevant images of the different scores describing how the nest quality assessment is conducted, possibly in the supplementary section. Although Figure 8 provides some information, the addition of a more detailed scale with pictures would be beneficial.

*We have now added supplemental Figure S5 which now shows representative images for different nest builds according to each score. This is referred to in the ‘Behavioural Metrics’ section of the Methods.*

Additionally, since the style of assembling the bedding material varies among mice, is it possible that the mothers in the Higgs study have differences despite all having the same background?

*This is not likely to be the case, and certainly does not explain the differences between WT and Magel2-null mice. Hopefully our revision of the nest-building score will clarify that the mutant mothers displayed nest building at level 1 or level 2, which results from not utilising the majority of the nest material and not building any structure resembling a nest. In contrast, WT controls all completed a level 3 nest.*

This paper by Ho-Shing and Dulac (2019, <https://doi.org/10.1016/j.cobeha.2018.08.008>) reports that loss of the paternally expressed Peg3 or Magel2 results in a reduction in hypothalamic oxytocin neurons. I am wondering if Magel2's effects on oxytocin releasing neurons might be responsible for some behavioural outcomes observed in this paper that seem to result from Magel2 defects in Gal+ neurons. For example, if there are fewer oxytocin releasing neurons, lactation might be impaired. In addition to keeping pups warm, crouching is also necessary in the lactation process. Observing a lower amount of crouching behaviour may result from Magel2 defects outside the Gal+ neurons identified in this study. What is the opinion of the authors?

*We agree with the reviewer that several other neuronal mechanisms could be in part responsible for the behavioural outcomes we have observed here. Oxytocin neurons from the Paraventricular nucleus project into the MPOA and regulate parenting so it is likely that these neurons would be having some kind of role. However, as the reviewer has pointed out, oxytocin neurons regulate specific facets of maternal behaviour such as crouching and so this mechanism is unlikely to explain all the behavioural deficits we have observed here, whereas the Gal+ neurons have broader impact on parenting. We have now included a sentence in the discussion raising awareness of alternative neuronal mechanisms to explain our deficits, with a specific focus on oxytocin neurons as the data from Magel2 and Peg3 show a loss of these neurons in mouse models and deficits in parenting behaviour.*

- *Line 417-420 - “Of note, a POA galanin mechanism does not prevent other mechanisms from acting and it is known that loss of Magel2 (43) and Peg3 (16) results in a loss of oxytocin neurons in mice, neurons which feed directly into the POA and the loss of which could be responsible for deficits in certain facets of parenting behaviour.”*

As the study revealed an improvement in performance during the second day of pup retrieval, I am wondering whether the same effect was observed in both mothers and fathers.

*In this set of experiments, we did not test mothers and fathers on a second occasion, so we do not know the answer for this specific cohort of mice. However, in a previous experiment (which can be found detailed in Appendix A4 of MJH’s thesis – https://orca.cardiff.ac.uk/id/eprint/158979/) we have performed second pregnancy assessments of WT Black6 mothers and fathers and found a modest improvement in fathers in their second attempt at the task while mothers tend to already be approaching a ceiling in their first pregnancy so although second time mothers are more consistent, their performance was not significantly better.*

I find the mix of terms used to refer to the experimental groups, such as "Magel2+/−", "Magel2+/p", "Magel2 knockout", and "Magel2 null", to be confusing. I recommend that the authors use a consistent terminology and simplify how they refer to the groups.

*We agree with the reviewer and have opted to use Magel2-null as the terminology to denote mice lacking the paternal allele of Magel2. When the groups are defined, we make it clear that Magel2-null is equivalent to Magel2+/p to ensure now confusion.*

Can the authors provide any information on the expression of Magel2 in the POA of Magel2m+/p− mice? Specifically, is there any expression from the maternal allele?

*We have not looked at Magel2 expression in the POA of the Magel2-null mice specifically. The knockout model we used is a constitutive deletion and so expression should be lost throughout the brain in the paternal knockouts –as is indicated by the new supplemental data (Figure S6). These new data also suggest that any “leaky” expression from the Magel2-null is negligible. However, there has been published evidence of stochastic leaky expression of the maternal allele of Necdin in Ndn paternal-knockout mice (*[*https://doi.org/10.1371/journal.pgen.1003752).*](https://doi.org/10.1371/journal.pgen.1003752).)

It may be useful to include a Venn diagram in Figure 1 to illustrate the overlap between genes such as Asb4, Calcr, Magel2, Ndn, Nap1l5, Peg3, Peg10, etc.

*This Venn diagram has been added to the figure as suggested by the reviewer and the figure legend updated accordingly*

Caption Figure 1: A) “a > 150%”: reads as a is greater than 150%

*The word ‘a’ has been removed to avoid confusion*

B) “elevated expression”: same as in A? expression level > 150%?

*In order to improve the robustness of any conclusions drawn from our investigation of imprinted gene enrichment, we chose to not rely on one scRNA-seq dataset at any given level of analysis. Here we used three separate datasets, which were processed individually in the original paper (Higgs et al., 2022, BMC Genomics). An unfortunate consequence of the fact that these different datasets need to be processed individually is that the criteria for elevated expression varies between datasets, and so cross-comparison means that in some instances the expression value was >100%.*

Figure 2A–B, 7A: missing scales

*Scales have now been added to these Figures*

Figure 2C–D:
Can you explain the meaning of the error bars in this graph? Perhaps, a better visualization would be a violin or box plot with a swarm plot overlay to show the distribution of values, which would be consistent with the presentation in Supplemental Figure S1.

*We agree with the reviewer than nonparametric data such as these may be better represented by Violin and swarm plots. However, we found that Violin and swarm plots were less informative given the high number of zeros in the data and the extremely high number of cells for each plot. Instead, we have opted to display the data using boxplots to better reflect the spread of the data. Figures 2C and 2D have been amended and better reflect the data.*

Figure 3, 4:
I suggest considering adding a panel that provides a description of the task.

*Although we agree that this would be a nice addition, since we already have a figure providing a visual description of the task, we have instead referred readers to Figure 8C to see an overview of the task with the legends for Figure 3,4 and 6. This is to save repetition and to avoid making the figure over-complicated.*

Figure 3A, 4A:
Was the test conducted with only three pups? The figure seems to show more.

*The test was conducted with only 3 pups. The figure was a stylised representation of a parent and their litter as a whole, but for clarity we have changed these images to only show 3 pups used in testing.*

Figure 3C–E, G, 4C–E, G, 6C–E, G:
It could be misleading to include the mothers and fathers who were unable to complete tasks C, D, or E or build the nest in panel G. Also in Figure 5C–D

*Whilst we agree that it will skew the pattern of the data, the failure to complete a task is a key indicator of poor parental behaviour. An imposed upper limit on a timed experiment always causes these problems, however, the limit of 60mins used here, is more than an adequate period of time to complete this task, as is indicated by the fact the majority of the control dams completed the task within 20 minutes. Moreover, we feel that to not include failures in the figures could be more misleading as it would suggest a much smaller N; this is particularly the case for the Magel2-null fathers. Consequently, we believe representing the failed animals in our figures is a true reflection of task performance.*

Figure 7: It would be helpful to have a clearer explanation of the term 'pup exposure' in the experimental scheme, and to provide more details about the different periods of isolation (1 hour) and pup exposure (30 minutes) mentioned in lines 608-622.

*We have updated Figure 7 to demonstrate the pup exposure process more clearly, we have also added a line in the figure legend to clearly describe the pup-exposure process as opposed to the control. Further detail has been added to lines 608-622 to further clarify the isolation and pup exposure periods.*

The legend needs to be adjusted such that the colour of the points matches that in the figure, where Control points are grey. However, it is a bit confusing that the female and male points are represented by light grey and grey, respectively, which can be easily mistaken for the Control points. Therefore, it may be better to replace the legend with "Fos-positive cells per 1000 Gal/Calcr-positive POA cells" because much of the differences observed are due to the varying numbers of Gal/Calcr-positive cells (as shown in Figure 7D). For example, the difference between Magel2-null and WT females is solely due to the differences in the number of Gal/Calcr-positive cells, and the same applies to the difference between Magel2-null and WT males.

*The legends in Figure 7B/C and Supplemental Figure S3 have been adjusted so that the point colour now matches the figure. However, to address the cross-over with light grey and grey being used for male and female, all points in Figure 7B and C are now monotone, since the bar colour does an effective job of distinguishing control from pup-exposed animals.*

*Although we agree that the data in 7D effectively explains the differences seen in Figure 7C, we believe it is important to present the results in this way so the reader can follow our chain of logic and see all the associated data regarding c-Fos response and Gal/Calcr cell numbers (rather than taking our word for it). It is also worth raising that the c-Fos difference seen in total (7B) are likely not only explained by differences in Gal/Calcr neuron cell number, as other cell types in the POA will be changing activity upon pup-exposure.*

Figures 7D–F - It is unusual that the bars in this figure are not divided as they were in Figures 7B-C, even though it is understood that no significant differences are expected.

*We agree with the reviewer that a more typical presentation of the data would have the bars in Figure D-F mirror those in B-C. However, the reason we have chosen to present the data this way is for clarity. As the reviewer notes, no significant difference of pup-exposure was observed when counting numbers of Gal-positive neurons, for example, and including this comparison within the figures adds unnecessary complexity.*

C–F) In previous figures, the WT bars were always presented before the Magel2-null bars. It might be better to follow the same pattern here and in corresponding supplemental figures.

*Figures 7C-F and Supplemental Figures S3 and S4 have been adjusted to show WT bars first followed by Magel2-null bars to reflect the previous figures*

Caption Figure 7: “5+ molecules”: Does it mean at least 5 molecules?

*Yes, this has been changed to ‘≥5 molecules’ for added clarity.*

Please maintain consistency in using the terminology "pup-directed behaviour" throughout the paper.

*All instances of the above term now appear as ‘pup-directed behaviour’*

L108–109: In contradiction Figure 1C seems to show that there are 10 upregulated imprinted genes in GABA10.

*It is not completely clear to us what this criticism refers to, but we think it is the fact that, although there are nine (not ten) highly expressed imprinted genes in the GABA10 cell cluster, imprinted genes as a group are not statistically over-represented in this cell cluster. Nevertheless, since the GABA10 population expressed very relevant marker genes (Gal, Calcr, Brs3), we included have included it in Figure 1C, as it is still pertinent. However, to improve clarity, this sentence has been reworded in response to reviewer 3’s comment.*

L109: 2B –> S2B

*Changed as suggested*

L118–119: language

*We were not 100% clear what this was refering too, but we believe it may relate to our use of ‘a number of’ which has now been changed to ‘many’*

Lines 185, 188, 193 and others: “F2, 62”: should probably be H(2) or F(2, 62)

*These examples and others have been corrected with brackets placed around the error terms*

Line 329: PDB (also used in Figures 3I–J and 4I–J) not defined in text (possibly in line 199)

*PDB was the abbreviation for pup-directed behaviour and is now defined in the text in its first appearance (Line 202) and in the Figure legends for which it is used in the axis label on some figures*

Line 276: check if W = 117 correct like in Line 274 but with different p-value, also check number of digits in rounding

*We have double-checked and this is correct. The existence of pairs in the data means we cannot compute an exact p-value which is why the two tests have the same W but differing p’s. We have adjusted all digit rounds to 2DP.*

Line 357–364: Unlike stated here, actually there is no effect of the Genotype on c-Fos expression (neither in Gal/Calcr-positive nor in Gal-positive nor in Calcr-positive cells), but rather on the number of cells expressing Gal and only subsequently c-Fos expression is influenced. The effect of the Genotype is only on the number of Gal/Calcr-positive, Gal-positive and Calcr-positive cells, but not on the c-Fos expression. The loss of c-Fos-positive cells is only for cells which are Gal-negative and Calcr-negative and only for females. Later the conclusion in Lines 377–378 states it correctly.

*We agree with the above and we agree it could be perhaps misleading to suggest a c-Fos deficits when in fact the deficit is in cell number and subsequently fewer cells are available to express c-Fos. However, we believe we do clearly state the interpretation that the reviewer has indicated on lines 377-378. Our reasoning for presenting the data in this way is that we feel it is informative for the reader to follow our chain of deduction in reaching the conclusion that a c-Fos reduction is, in part, the result of fewer cells rather than a loss of cell activity. This level of granularity is also made possible by our use of RNAscope, which is quantitative, rather than conventional in situ hybridisation.*

Line 393–394: “Magel2-null females had reduced Gal expression in the POA and a reduced c-Fos response in the POA upon exposure to pups”:
◦ structure of sentence may be misunderstood: “reduced Gal expression upon exposure to pups”

◦ It seems that the sentence suggests that exposure to pups resulted in a decrease in c-Fos response, when in fact, the opposite is true. Perhaps the intention was to say that the increase in c-Fos expression is not as significant in Magel2-null females as it is in WT females. It would be helpful to clarify this in relation to Figure 7B (left). The figure shows an increase in c-Fos expression for both WT and Magel2-null females, but it would be beneficial to know the p-value for this difference.

*This sentence has been restructured to avoid the misunderstanding highlighted and to indicate that the reduced c-Fos response was compared to WT – “Magel2-null females had a less significant c-Fos response in the POA upon exposure to pups compared to WT’s and a general reduction in Gal expression in the POA”*

**Reviewer #3:**

“Did the authors assess the binding specificity of the Magel2 probe in the null brains? These data would provide a control that they are interrogating the correct molecule in the RNAscope experiments and should be including in supplementary information if available”

*No we did not assess the binding specificity of the probe, but this probe, which is commercially available (ACD Cat #535901) has been previously validated and published (*[*https://doi.org/10.1038/s41380-021-01227-6*](https://doi.org/10.1038/s41380-021-01227-6)*). We have made this clear in the Materials and Methods section.*

Line 107 - this sentence does not make sense “Of interest were, GABA13 (Gal, Slc18a2 and Th) which showed enrichment for imprinted genes and GABA10 (Gal, Calcr and Brs3) (Supplemental Table 2B) but was not enriched for imprinted genes.”

*This has been changed to “Of interest were two galanin-enriched neuron types, GABA13 (Gal, Slc18a2 and Th) and GABA10 (Gal, Calcr and Brs3) (Supplemental Table 2B).”*

Figure 1 - It should be made clear what the genes listed in parentheses after each cell subtype are - I assume they are the defining neuronal markers for that cell type.

*The reviewer is correct in that these are the neuronal markers for that cell type, we have now amended the legend of Figure 1 to make this clear.*

- ***“Figure 1.*** *Imprinted genes upregulated in galanin neuron subpopulations. Imprinted genes are presented in red and blue which indicates their parent-of-origin expression; blue – PEGs, red – MEGs. Genes in black and present in parentheses are the neuronal markers for that cell type.”*

Line 184 - The time taken for to complete the task... The “for” should be removed.

*Changed as suggested*

For Supplementary Table 4B - Chapter 4 is mentioned in the legend. This should be removed.

*This has now been removed, the legend now reads:*

- ***Supplemental Table S4B.*** *Proportion of different cell types with varying numbers of Magel2 reads and the corresponding H-scores. H-scores are calculated from weighted calculation of proportions using the guidance from ACD Bio-techne (SOP 45-006)*
